# The Evaluation of Lymphadenopathy in a Resource-Limited Setting

**DOI:** 10.7759/cureus.30623

**Published:** 2022-10-24

**Authors:** Mohammed Ali, Ahmed Elhatw, Mai Hegazy, Hossam Albeyoumi, Noha Sakr, Ashrakat M Deyab, Ammar Yasser Soliman, Ebram Said, Ahmad Samir Elbehwashy, Mahmoud Nassar, Mostafa Alfishawy

**Affiliations:** 1 General Practice, Al-Azhar University, Cairo, EGY; 2 Radiology, Cairo University, Cairo, EGY; 3 General Surgery, Cairo University, Cairo, EGY; 4 Cardiovascular Medicine, Cairo University, Cairo, EGY; 5 Oncology, Ain Shams University, Cairo, EGY; 6 Pediatrics, Cairo University, Cairo, EGY; 7 Internal Medicine, Misr University for Science & Technology (MUST), Cairo, EGY; 8 Cardiology, Mayo Clinic, Arizona, USA; 9 Infectious Diseases, National Hepatology and Tropical Medicine Research Institute (NHTMRI), Giza, EGY; 10 Internal Medicine, Icahn School of Medicine at Mount Sinai/New York City (NYC) Health + Hospitals/Queens, New York, USA; 11 Infectious Diseases, Infectious Diseases Consultants and Academic Researchers of Egypt (IDCARE), Cairo, EGY

**Keywords:** resource-limited setting, unilateral hilar lymphadenopathy, reactive lymphadenopathy, etiology and treatment, clinical evaluation, peripheral lymphadenopathy, lymph node lesion

## Abstract

Lymphadenopathy (LAP) refers to abnormalities in the size or consistency of lymph nodes. A wide range of etiologies contributes to the difficulty in diagnosing LAP, from mild self-limited viral infections to grave autoimmune diseases and malignancies. Detailed history-taking and a thorough physical examination are essential. Some physicians in developing countries may consider therapeutic testing. Certain groups may require additional evaluations and special treatment. When treating LAP, the etiology is targeted, but corticosteroids should not be administered before a complete diagnosis has been established due to their ability to mask the histological diagnosis of lymphoma and malignancy. This review aims to provide more straightforward and affordable methods available in almost all healthcare settings, especially those with limited resources.

## Introduction and background

Lymphadenopathy (LAP) refers to abnormal growth or consistency of lymph nodes. Lymph nodes typically measure no more than 1 cm in diameter, but exceptions include inguinal lymph nodes, which may reach 1.5 cm, and epitrochlear nodes, which are typically 0.5 cm [1]. Lymph nodes of children between the ages of two and 10 are generally more prominent. Lymph nodes larger than 2 cm may indicate malignancy such as lymphoma or granulomatous diseases such as tuberculosis or cat scratch disease [2].

The etiology of LAP can range from self-limited infections common to younger adults to malignancies prevalent in older adults. Different geographical areas can also have various etiologies. Some African regions are prone to cervical LAP due to tuberculosis [3]. Nonspecific reactive lymph node changes are LAP’s most common benign etiology [4]. A comprehensive medical history can significantly facilitate the diagnosis of many of these cases without imposing a cumbersome burden on the healthcare system through costly tests and biopsies.

Guidelines for diagnosing LAP call for investigations that are too expensive to be routinely implemented in most third-world healthcare institutions. The median cost of inpatient surgical biopsies ($29,988) was higher than that of outpatient percutaneous biopsies ($1,028). Repeat biopsies increased costs by 40%-80%, with complications accounting for 13% of the costs [5]. Therefore, we present in this paper more straightforward methods that are available and affordable in almost all healthcare settings.

## Review

Medical history

Thorough history-taking is essential in determining the etiology behind LAP. Most cases of LAP in children are benign or caused by infections, and nearly half of all cases occur in healthy individuals [6]. A LAP without progression in size lasting less than two weeks or more than a year is unlikely to be neoplastic in children or adults. Hodgkin’s lymphoma and indolent non-Hodgkin’s lymphoma (NHL) are exceptions to this rule [7].

Medications

The history-taking process should include an inquiry about certain drugs associated with LAP. Identifying the drug that is causing LAP will save time and money, as well as decrease patient anxiety. The following medication categories are associated with LAD: cardiovascular medications, central nervous system medications, antibiotics, and anti-gout medications (Table 1) [7].

**Table 1:** Medications associated with LAP LAP: lymphadenopathy

Category	Medication
Cardiovascular medications	Quinidine, hydralazine, captopril, and atenolol
Central nervous system medications	Phenytoin and primidone
Antibiotics	Penicillin and trimethoprim/sulfamethoxazole
Anti-gout	Allopurinol

Associated Symptoms

Autoimmune diseases may be associated with LAP, such as rheumatoid arthritis, systemic lupus erythematosus (SLE), and dermatomyositis. A history of rash, arthralgia, myopathy, or anemia suggests an autoimmune etiology [7]. On the other hand, an infectious etiology should be suspected if there are associated constitutional symptoms such as fever, headache, malaise, or fatigue [8]. Unexplained weight loss of greater than 10% of the body weight during six months, accompanied by fever and night sweats, is suggestive of Hodgkin’s lymphoma or non-Hodgkin’s lymphoma (NHL) or tuberculosis [9]. Generalized pruritus is also associated with Hodgkin’s lymphoma and NHL [7].

Environmental Exposures

Several factors have been linked to LAP, including prolonged exposure to ultraviolet (UV) radiation, smoking or drinking alcoholic beverages, exposure to undercooked meat, unprotected sexual encounters, and contact with cats, rabbits, or sheep, as shown in Table 2. Vaccines have also been reported to cause LAP (Table 3).

**Table 2:** History of exposures suggesting specific causes of LAP UV: ultraviolet, LAP: lymphadenopathy, HIV: human immunodeficiency virus

Exposure	Disease
Prolonged exposure to UV radiation, smoking, or alcoholic drinks	Malignancies
Contact with cats	Cat scratch disease and toxoplasmosis [10]
Contact with rabbits or sheep	Anthrax, brucellosis, and tularemia
Exposure to undercooked meat	Anthrax, brucellosis, and toxoplasmosis [11]
Unprotected sexual encounters	Chancroid, HIV, lymphogranuloma venereum, and syphilis [7]

**Table 3:** Vaccinations associated with LAP [12,13] LAP: lymphadenopathy, COVID-19: coronavirus disease 2019, BCG: Bacille Calmette-Guérin, HPV: human papillomavirus

Vaccine
H1N1 influenza vaccine
Smallpox vaccine
BCG vaccine
HPV vaccine
Pfizer-BioNTech COVID-19 vaccine
Moderna COVID-19 vaccine
Oxford-AstraZeneca COVID-19 vaccine

Physical examination

It is crucial to determine whether LAP is localized or generalized [1]. Inspect the skin for erythema, masses, or skin infections. Examine the head and neck for rashes, scalp lesions, conjunctivitis, nasal obstruction or sinusitis, dental caries, or pharyngitis. Regardless of whether the lymph node is unilateral, bilateral, or generalized, it should be examined. Identify the characteristics of the lymph node, including its fluctuation and fixation. Certain clues in the examination could aid in the diagnosis (Table 4).

**Table 4:** Clues to diagnosis

Area	Examination	
Skin	Inspect for trauma or erythema or other masses or skin infection as a viral rash, a cat scratch, or purulence [1,14].	
Head and neck	Viral or bacterial rashes, scalp lesions such as tinea capitis, conjunctivitis, nasal obstruction or sinusitis, dental caries, or pharyngitis.	
Abdomen	Look for other masses and assess for hepatomegaly or splenomegaly.	
Lymph nodes	All lymph nodes and their catchment areas should be examined, whether unilateral, bilateral, or generalized [15,16].	
	Area	Clues
Location	Unilateral lymphadenopathy	Lymphoma
	Bilateral, soft, and mobile	Viral infection
	Unilateral or bilateral, hard, and immobile	Bacterial infection
	Supraclavicular lymph node	Malignancy (patients of 40 years of age suggests a 90% risk)
	Left supraclavicular nodes	Gastric carcinoma
	Right supraclavicular nodes	Intrathoracic neoplasm
	Submandibular and posterior cervical nodes	Mononucleosis [17,18]
	Hard nodes	Malignancy
	Soft and firm	Infiltration by inflammation and leukemia [1]
Fluctuance	Bacterial infection, especially with *Staphylococcus aureus*, causes fluctuance and abscess formation [18].	
Fixation	Free, mobile, and no adhesion to adjacent structure and skin	Normal lymph nodes
	Adhesion	Infiltration of inflammatory cells of infection or leukemia
	Matted	Tuberculosis [16]

Investigations

A thorough history and physical examination usually reveal the underlying cause of LAP. A diagnostic approach will assist in reaching a diagnosis. However, some cases may not require further investigation, such as conjunctivitis, pharyngitis, or focal infections with regional lymph node involvement. This is also true for the patient with an apparent viral upper respiratory tract infection with cervical lymphadenopathy.

Laboratory Investigations

Additional testing may be required if the history and examination suggest an atypical infection, malignancy, or autoimmune disease. A complete blood count (CBC) with differential may provide clues to the diagnosis by identifying the causes of anemia, neutrophilia, lymphocytosis, pancytopenia, thrombocytosis, or blast cells (Table 5).

**Table 5:** CBC with differential findings CBC: complete blood count, WBCs: white blood cells, HIV: human immunodeficiency virus, TB: tuberculosis, CMV: cytomegalovirus, EBV: Epstein-Barr virus

Finding	Suggestive cause
Anemia	Systemic lupus and leukemia
Neutrophilia	Acute bacterial infection (a leukemoid reaction (WBCs > 50,000/mm^3^) suggests a severe infection or, uncommonly, myeloproliferative disease, e.g., chronic myelocytic leukemia)
Lymphocytosis	Leukemia, CMV, and TB (lymphocytosis with >50% leukocytes and 10% atypical lymphocytes with positive EBV serology testing is a classic finding in EBV infection)
Pancytopenia	Systemic lupus, HIV, and leukemia
Thrombocytosis	Acute-phase Kawasaki disease
Blast cells	Leukemia

If history and examination suggest an autoimmune etiology, an autoimmune workup should be conducted, including antinuclear antibodies (ANA), double-stranded DNA (dsDNA) antibodies, C-reactive protein (CRP), erythrocyte sedimentation rate (ESR), rheumatoid factor, and complement levels. If the diagnosis remains uncertain, second-tier tests may be indicated (Table 6). Observing localized LAP for four weeks is possible if malignancy is considered unlikely based on the history and physical examination.

**Table 6:** Second-tier tests PCR: polymerase chain reaction, PPD: purified protein derivative, RPR: rapid plasma regain, VDRL: Venereal Disease Research Laboratory, HIV: human immunodeficiency virus, TB: tuberculosis, CMV: cytomegalovirus, EBV: Epstein-Barr virus, IGRAs: interferon-gamma release assays

Suggested diagnosis	Diagnostic test
Cat scratch disease	Serology and PCR
Tuberculosis	PPD skin test, IGRAs, sputum culture, and chest X-ray
Toxoplasmosis	Serology
HIV	HIV1/HIV2 immune essay
Mononucleosis	Monospot test and EBV serology
Brucellosis	Blood culture, serology, and PCR
Rubella	Serology
CMV	CMV antibody latex and CMV PCR
Pharyngitis	Throat swab and culture
Tularemia	Blood culture and serology
Syphilis	Syphilis antibodies, RPR, and VDRL

Imaging

Imaging can determine the size and distribution of lymph nodes more accurately than physical examination and assess the involvement of surrounding structures. Although imaging can provide diagnostic clues, it cannot replace a biopsy. For children up to 14 years of age with cervical lymphadenopathy, ultrasound should be the first imaging method of choice. For patients over the age of 14 years, a computed tomography (CT) scan should be the investigation of choice. Other imaging modalities can be utilized to assess and guide the diagnosis of LAP, such as chest X-ray (CXR), magnetic resonance imaging (MRI), color Doppler ultrasounds, and contrast-enhanced ultrasonography. Chest X-rays (CXR) have a high sensitivity for pulmonary tuberculosis but low specificity.

Pathology

**Figure 1:**
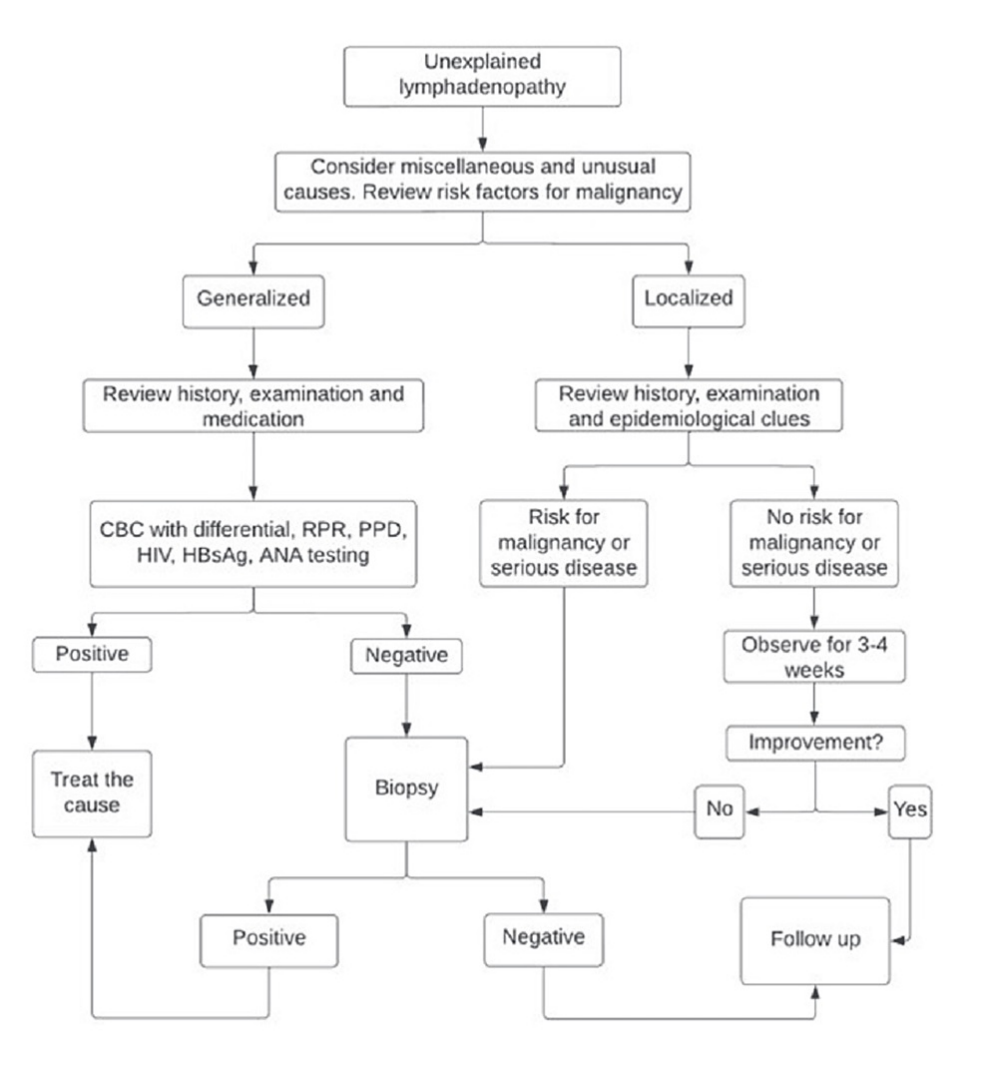
Pathway to diagnosis to manage unexplained LAP CBC: complete blood count, WBCs: White blood cells, HBsAg: hepatitis B surface antigen, HIV: human immunodeficiency virus, PPD: purified protein derivative, RPR: rapid plasma regain, HIV: human immunodeficiency virus, ANA: antinuclear antibody

The gold standard for evaluating LAP is tissue diagnosis [1]. It provides histopathological evaluation and microbiologic diagnosis through culture or molecular diagnostics of specimens. The following factors help determine the need for a lymph node biopsy: age of more than 40 years, supraclavicular lymph node enlargement, nodal diameter greater than 2.25 cm, firm, hard texture, and lack of pain [1]. The following algorithm is used to manage unexplained lymphadenopathy (Figure 1). A comparison of tissue biopsy methods such as fine needle aspiration cytology (FNAC), core needle biopsy, and excisional biopsy is presented in Table 7.

**Table 7:** Comparison of tissue biopsy methods FNAC: fine needle aspiration cytology

	Strengths	Limitations
FNAC	It provides maximum sensitivity and specificity for detecting metastatic cancers [19], as preserving lymph node architecture is not essential for diagnosis.	Inadequate specimens, high rate of false-negative diagnoses in Hodgkin’s disease, and incomplete classification of non-Hodgkin’s lymphoma [20].
Core needle biopsy	It provides tissue for special studies and some information on nodal architecture, has relatively low morbidity, and is an inexpensive alternative to open biopsy when an intact node is not easily accessible [21].	Its strength for diagnosing lymphoma is controversial, and excisional biopsy of enlarged lymph nodes is the gold standard procedure [1].
Excisional biopsy	It allows histological examination of intact tissues. It informs the presence of malignant cells and microorganisms. It informs nodal architecture, aiding the diagnosis of lymphomas [16].	The most abnormal node is selected. If no single node predominates, the selection of the lymph node should be in descending order: supraclavicular, neck, axillary, and groin. This order avoids nonspecific results and injury to neurovascular structures. The pathologist should be informed in advance to prepare for the proper smears, stains, and cultures [16].

Special groups

Children

Children are susceptible to lymphadenopathy when their immune systems are activated by environmental antigens and common organisms [22]. Identifying the cause of localized lymphadenopathy in children may be possible based on the location of the lesions. Anterior cervical lymph nodes are enlarged in a variety of infections of the head and neck, as well as in systemic infections such as Epstein-Barr virus (EBV), toxoplasmosis, and cytomegalovirus. Only a quarter of children with cervical lymphadenopathy have a serious disease such as mycobacteria. The upper posterior group is rarely associated with significant diseases in children [23,24].

The supraclavicular group is associated with an increased risk of malignancy in children [23,25]. Right supraclavicular lymph nodes are associated with malignant mediastinal lymph nodes. Virchow’s lymph node is associated with intra-abdominal malignancies, usually metastatic diseases such as neuroblastoma and lymphoma [26]. The axillary region is predominant in Bacille Calmette-Guérin (BCG) vaccine infections, *Mycobacterium tuberculosis* infections, and cat scratch disease [23,27]. The inguinal area is not specific to children unless it is larger than 3 cm [28,29]. Epitrochlear lymph nodes are often associated with pathological causes in children, and the differential diagnosis includes infections of the forearm or hand, leukemia, lymphoma, and atypical mycobacterial infections [18].

Immunocompromised Individuals

Due to the possibility of disease progression, laboratory tests and biopsies are often required to establish a diagnosis. Lymphadenopathies in immunocompromised patients are classified as neoplastic and non-neoplastic [30]. Non-neoplastic lymphadenopathy is primarily caused by infectious agents, such as bacteria, fungi, and parasites. In the immunocompromised, non-neoplastic lymphadenopathy may also be caused by reactive hyperplasia. Neoplastic causes include lymphomas, Kaposi sarcoma, and metastatic cancers.

Pregnancy

It is essential to exclude the following conditions from the differential diagnosis of lymphadenopathy during pregnancy [31,32]. Studies have shown that flare-ups of SLE are more likely to occur during pregnancy [33]. Lymphadenopathy in patients with SLE is not associated with a risk, and its presence correlates with a high level of disease activity [34]. The majority of patients with sarcoidosis are female, and most are diagnosed during their reproductive years [35]. Toxoplasmosis usually affects the posterior cervical group [36]. Cytomegalovirus should also be considered in the differential diagnosis.

Therapeutic testing and limited resources

A course of empiric antibiotics should be considered for patients with LAP if they have constitutional symptoms, unilateral LAP, erythema, and tenderness or if the lymph nodes are larger than 2-3 cm in size. Since *Staphylococcus aureus* and group A streptococcus are the most common organisms, a 10-day course of either cephalexin, amoxicillin/clavulanate, or clindamycin is recommended. Corticosteroids should be avoided as they can mask the histological diagnosis of lymphoma and malignancy.

We recommend the following if the initial antibiotic trial fails in a setting where resources are limited. The addition of clindamycin to a patient’s treatment regimen should be considered when community-associated methicillin-resistant *Staphylococcus aureus* (CA-MRSA) is prevalent. Doxycycline should be added to the treatment program in areas where *Rickettsia* infections are more prevalent. Adding azithromycin may be necessary if there has been contact with cats that suggests cat scratch disease. The use of pyrimethamine and sulfadiazine, along with leucovorin, is recommended when toxoplasmosis is suspected following contact with cats. In areas with a high prevalence of tuberculosis, therapeutic testing with antituberculosis drugs may be necessary. Therapeutic testing with antituberculosis drugs for two weeks may be warranted.

## Conclusions

Identifying the etiology of lymphadenopathy can be challenging due to the wide variety of etiologies and the need for extensive investigations to reach an accurate diagnosis. Developing countries may not have access to some of these investigations, so therapeutic testing should be offered. Also, special attention should be paid to children, pregnant women, and patients with immunocompromised conditions.
